# The Interplay between *Mycobacterium tuberculosis* and Human Microbiome

**DOI:** 10.3390/clinpract14010017

**Published:** 2024-01-24

**Authors:** Michelle Nguyen, Phillip Ahn, John Dawi, Areg Gargaloyan, Anthony Kiriaki, Tiffany Shou, Kevin Wu, Kian Yazdan, Vishwanath Venketaraman

**Affiliations:** College of Osteopathic Medicine of the Pacific, Western University of Health Sciences, Pomona, CA 91766, USA; michelle.nguyen2@westernu.edu (M.N.); phillip.ahn@westernu.edu (P.A.); john.dawi@westernu.edu (J.D.); areg.gargaloyan@westernu.edu (A.G.); anthony.kiriaki@westernu.edu (A.K.); tiffany.shou@westernu.edu (T.S.); kevin.wu@westernu.edu (K.W.); kian.yazdan@westernu.edu (K.Y.)

**Keywords:** *Mycobacterium tuberculosis*, tuberculosis, microbiome, dysbiosis

## Abstract

Tuberculosis (TB), a respiratory disease caused by *Mycobacterium tuberculosis* (Mtb), is a significant cause of mortality worldwide. The lung, a breeding ground for Mtb, was once thought to be a sterile environment, but has now been found to host its own profile of microbes. These microbes are critical in the development of the host immune system and can produce metabolites that aid in host defense against various pathogens. Mtb infection as well as antibiotics can shift the microbial profile, causing dysbiosis and dampening the host immune response. Additionally, increasing cases of drug resistant TB have impacted the success rates of the traditional therapies of isoniazid, rifampin, pyrazinamide, and ethambutol. Recent years have produced tremendous research into the human microbiome and its role in contributing to or attenuating disease processes. Potential treatments aimed at altering the gut-lung bacterial axis may offer promising results against drug resistant TB and help mitigate the effects of TB.

## 1. Introduction to Tuberculosis: Incidence and Significance of the Problem 

Tuberculosis (TB) is a highly infectious airborne disease caused by the bacilli *Mycobacterium tuberculosis* (Mtb). It ranks as the thirteenth leading cause of death worldwide and the second leading cause of death from a singular infectious agent behind COVID-19 [1]. According to the Global Tuberculosis Report 2022 by the World Health Organization (WHO), an estimated 10.6 million people globally were ill with TB in 2021, reflecting a 4.5% increase compared to that in 2020 [1]. Additionally, the incidence of TB cases increased from 2020 to 2021 by roughly 3.6% [1]. Of all TB cases in 2021, 6.7% of cases were co-morbid with acquired immune deficiency syndrome (AIDS), which thereby presents as a risk factor to TB acquisition along with factors such as poverty and indoor air pollution [1,2,3]. To emphasize the great impact TB has globally, notable geographic hotspots of the 2021 TB cases include South-East Asia (45%), Africa (23%), and the Western Pacific (18%) [1]. Death numbers have grown as well, with 1.6 million deaths occurring in 2021 in comparison to 1.5 million deaths in 2020 [1]. TB therefore continues to pose a significant health risk worldwide.

During the COVID-19 pandemic, TB resources were difficult to access by affected persons. The WHO reported decreases in TB diagnoses during the pandemic, hinting at the resultant underlying increase in undiagnosed TB cases. The WHO’s End TB strategy, adopted by the World Health Assembly in 2014, aims to decrease TB incidence by 80% by 2023. However, the undiagnosed TB cases as well as a rise in drug resistant TB cases complicates this goal.

The current regime for TB treatment includes combinations of rifampicin, isoniazid, pyrazinamide, and ethambutol; however, medication adherence is complicated by long durations of treatment varying from 3 months to 9 months [4,5,6]. TB program campaigns additionally face difficulties with inadequate human resources, disease monitoring, case reporting, and drug supplies [6]. Multi-drug resistant (MDR) and extensively drug resistant (XDR) TB strains pose an added threat, with a 6.4% increase in MDR/XDR-TB cases between 2020 to 2021 [1]. Drug resistance expectantly impacts treatment success; in 2019, treatment success for drug resistant strains was 60%, which pales in comparison to an overall TB treatment success rate of 86% [1]. There is currently only one licensed vaccine for TB disease prevention, the Bacillus Calmette-Guérin (BCG) vaccine. However, the BCG vaccine is generally used and recommended only in children; therefore, there is no licensed TB vaccine for adults [1].

TB currently ranks among the top three threats to global public health alongside malaria and AIDS [7]. With the growing number of TB cases and fatalities, drug resistant strains, and barriers to treatment programs, more research is needed in available and sustainable treatment options as well as preventative medicine [7]. Therapies targeting the human microbiome may present as a potential avenue to attenuating TB mortality as the gut microbiome can modulate the host immune system both locally and systemically [8].

## 2. Host Immune Response against *Mycobacterium tuberculosis*

To further understand how the microbiome can impact TB progression, we must first review the pathogenesis of and host immune response against Mtb. The pathogenesis begins when respiratory droplets from an individual with active TB are transmitted via inhalation of airborne particles that travel down the respiratory tract and enter the lung alveoli. Mtb bacilli are then phagocytosed by alveolar macrophages and are able to multiply intracellularly, evading the innate immune response. The alveolar macrophages then die and the bacteria are released. This dissemination of Mtb elicits a host adaptive immune response [9]. In a study by Lu et al., CD8+ T cells from lung samples of Mtb-infected major histocompatibility complex (MHC)-II knockout (KO) mice and Mtb-infected wildtype (WT) mice were analyzed [10]. MHC-II KO mice displayed higher Mtb burden in the lungs and died shortly after infection compared to their controls [10]. In addition to demonstrating how compromised CD8+ T cell function contributes to decreased immunity, Lu et al. also showed that CD4+ T cells are imperative in controlling Mtb infection and promoting host survival by enhancing the function of CD8+ T cells [10]. Additionally, other studies have illustrated that the cytokines IFN-γ and TNF-α produced by T lymphocytes are crucial in controlling Mtb infection by inducing pathogen uptake by macrophages and killing via reactive nitrogen intermediates [11,12]. However, the activation of other cytokines such as IFN-α, IFN-β, IL-1β, IL-6, and IL-12 may contribute to the inflammation and destruction of lung tissue that is seen in TB patients [13,14].

**Figure 1:**
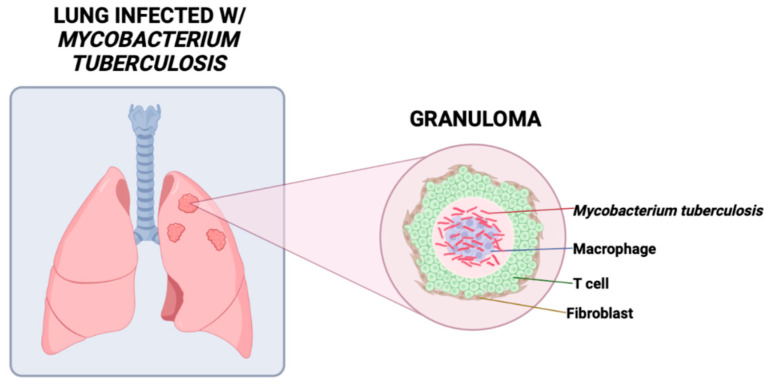
A granuloma, the hallmark of TB, is an aggregation of fibroblasts, macrophages, lymphocytes, and other immune cells that limits spread of *Mycobacterium tuberculosis* and provides an acidic environment optimal for proliferation.

One of the hallmarks of pulmonary TB in humans is the formation of a granuloma, a defensive inflammatory mechanism by which the body can sequester pathogens (Figure 1). The formation of a granuloma in addition to the surrounding influx of macrophages and other immune cells such as T lymphocytes, B lymphocytes, neutrophils, dendritic cells, and fibroblasts occur shortly after initial Mtb infection to help fight off the pathogen [15]. Additionally, T helper (Th)17 cells play a protective role through secreting IL-17, signaling for further chemokine secretion, and recruiting neutrophil to the infection site [16,17]. Previous research has shown that mice deficient in IL-17 were unable to progress granulomas from nascent to mature [18]. This suggests that IL-17 plays a crucial role in granuloma maturation and defending against mycobacterial infections as granulomas play a critical role in preventing the lymphatic and vascular dissemination of Mtb to nearby pulmonary lymph nodes and other extrapulmonary tissues [18,19]. However, although the alveolar macrophages and granulomas work to protect the host from Mtb, the containment of dormant bacteria within intracellular compartments allow Mtb to persist in the latent stage of infection for decades [20,21].

Primary TB is defined as the clinical manifestations upon initial contact with the Mtb organism. Before entering the latent stage of infection, primary TB is characterized by the formation of a Ghon complex localized to the middle portion of the lungs. Latent TB arises when the Ghon complex progresses into a latent stage [22]. In latent TB infection, the affected individual is a carrier of Mtb antigens within their system; however, there is no manifestation of clinical symptoms [23]. In active TB, the most common physical findings are a chronic cough, hemoptysis, weight loss, low-grade fever, and night sweats [22]. Whether secondary TB is caused by reinfection or by reactivation, the clinical presentation is more severe than primary TB in the degree of tissue reaction and hypersensitivity [24].

Active TB may develop from reinfection with Mtb or from reactivation of a latent TB infection [9,25]. In reactivated TB, host immune cells fail to suppress the active replication of Mtb, and the disease manifests as host tissue becomes damaged by excessive inflammatory responses that cause necrosis and cavitation [26,27]. There are many risk factors that promote the occurrence of reactivated TB, including older age, malnutrition, and underlying medical conditions that compromise the host immune system. Cancer, diabetes mellitus (DM), and immunosuppressive therapies are all conditions that lead to an immunocompromised host [25,28]. Among all risk factors, human immunodeficiency virus (HIV) infection is the most prominent condition leading to reactivated TB due to depletion of CD4+ T cells and their protective effects [29]. In a study of patients with active TB, patients with latent TB, patients who had previously recovered from TB, and healthy controls, it was discovered that there were significantly decreased levels of T lymphocytes in patients with active TB [30]. This suggests that depleted T cells lead to a more vulnerable host and progressing TB disease course.

Because immune function is pliable and susceptible to changes by the human microbiota, insight into the effects of dysbiosis and inflammatory processes can help to develop more effective Mtb treatments and therapies in the future.

## 3. The Human Microbiome

The gut microbiota is a complex system that consists of trillions of commensal microorganisms that mainly reside in the large intestine of the gastrointestinal tract [31]. The collective genes of the microorganisms, which include fungi, archaea, parasites, viruses, bacteria, etc., are referred to as the ‘microbiome’. More specifically, the gut bacterial flora is composed of species from seven main different phyla: Bacteroidetes, Firmicutes, Actinobacteria, Fusobacteria, Verrucomicrobia, Cyanobacteria, and Proteobacteria. However, a vast majority of these bacteria belong to the Bacteroidetes and Firmicutes phyla, which emerging studies have considered as a positive predictive marker for health and disease [31,32,33,34,35].

**Figure 2:**
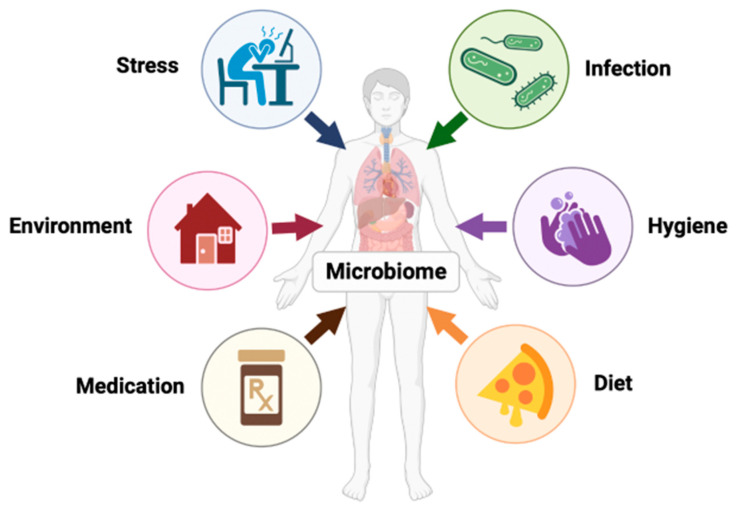
The human microbiome is dynamic and influenced by factors such as stress, infection, hygiene, age, genetics, geographics, etc.

Recent studies suggest that the intestinal microbiome evolves in parallel with the host throughout life, with most changes occurring in the first few years of life [36]. This instability in gut diversity eventually subsides and resembles an adult’s microbiome after 3–5 years of age [31]. These compositional changes remain the same throughout adulthood unless influenced by genetic and environmental factors such as long-term dietary habits, living environment, antibiotic use, etc. (Figure 2) [35,36]. Therefore, the human microbiome is susceptible to both beneficial and harmful changes throughout life.

Current data in gut microbiome science has now determined that diet is the primary driver of microbial diversity, which has direct impacts on adaptive and innate immunities of the host [35,36,37]. Poor diet leads to imbalances in the microbiome, or dysbiosis, which can lead to increased susceptibility to opportunistic infections and non-communicable chronic diseases (NCCDs) [37,38]. Dysbiosis compromises the host’s immune system due to the microbiome’s involvement in mechanisms that include nutrient absorption and processing, inflammatory pathways, and metabolite production [37,38,39].

The immunomodulatory effects of gut microbiota can occur locally and can also be disseminated to other organs, including the lungs. There is increased evidence that there is a microbial interaction between the respiratory and gastrointestinal tracts, which is now referred to as the ‘gut-lung axis’ [35,36,40]. This axis is a bi-directional system where changes in intestinal microbiota can cause greater susceptibility to pulmonary infections, and pulmonary infections have been seen to modulate microbial diversity [35,36,40]. Here, we will discuss the scientific evidence that shows possible associations between gut microbiota homeostasis and Mtb pathogenesis, therapy, health outcomes, and post-treatment outcomes.

Studies have found that (1) Mtb infection shifts and lowers microbial diversity, resulting in decreased Firmicutes, Bacteroidetes, and short-chain fatty acid (SCFA) producing bacteria as well as increased Actinobacteria and Proteobacteria [41,42,43,44,45]; (2) there is increased susceptibility to re-infection of Mtb due to depleted antigens for T cells in the gut microbiota [41,46,47]; and (3) prolonged antibiotic treatment disrupts microbiota composition [40,41,48,49].

## 4. The Microbiome on Immunity

The microbiome plays a critical role in constructing the host immune system. Unconventional T cells, such as mucosal-associated invariant T cells (MAIT), that are present in mucosal tissue in the lungs and gut play a role in the defense and control of Mtb infection [43,46]. The development and maturation of these unconventional T cells depend on the presence of the host microbiota [43,46]. Mice with antibiotic-altered microbiota as well as controlled mice without antibiotic alterations were infected by Mtb in a study conducted by Dumas et al. and a decline in MAIT cell quantity and a consequent decrease in IL-17A production were observed in mice with antibiotic-altered microbiota [46]. IL-17 secretion during mycobacterial infections has been associated with anti-Mtb immunity due to signaling for further chemokine secretion, recruiting neutrophils to the site of infection, and assisting in granuloma maturation to control mycobacterial infection [16,17,18]. It was found that early Mtb lung colonization in mice was linked to impaired MAIT cell functions and microbiota dysbiosis [46]. However, dysbiosis attenuation through inoculating microbiota in the antibiotic-treated mice positively affected MAIT cell proliferation [46]. This highlights the importance of a healthy microbiota and the role for potential microbial-altering therapies for proper pulmonary MAIT cell function and early control of Mtb growth in the lungs through IL-17A production [46]. It has also been shown that induction of MAIT cell expression through the administration of 5-(2-oxopropylideneamino)-6-d-ribitylaminouracil (5-OP-RU), a microbial riboflavin-derived antigen, reduces Mtb loads during chronic pulmonary infection, reinforcing the importance of MAIT cells in and suggesting an additional potential strategy for TB control [43]. However, Th17 and IL-17 may only be effective in an initial response to Mtb as their long-term presence is correlated with inducing autoimmune disorders [16]. Thus, further research in this area is needed to understand the role of specific microbiota species in mediating the protection against Mtb infection through MAIT cells, to examine if the prolonged production of IL-17 has any effect on the susceptibility of patients to Mtb, and to determine to what extent does this response begin to negatively impact the disease process rather than control it.

Furthermore, Dumas et al. showed that the dysbiosis of mice with early Mtb lung colonization was characterized by reduced Bacteroidetes and Firmicutes and increased Proteobacteria [46]. Cytophaga-flavobacter-bacteriodetes bacteria (CFB) is associated with the differentiation of Th17 cells and favors this process in the small intestines more than in other parts of the GI system. Antibiotic treatments can inhibit the growth of CFBs and potentially confer Mtb susceptibility [50]. A study conducted by Yang et al. explores the role of *Bacteroides fragilis*, which was found to be decreased in mice infected with Mtb, in influencing expression of a type of non-coding RNA (ncRNA) called long non-coding RNA (lncRNA) [51]. Non-coding RNAs have been implicated in modulating host-microbe interaction and related pathologies like obesity [52]. Various lncRNAs were described as having roles in regulating inflammatory mediators [53], and it was found by Yang et al. that gut dysbiosis disturbed proper immune functioning, leading to repressed cytokines such as IFN-γ [51]. Additionally, *B. fragilis* induces increased levels of lncRNA, which in turn increases the expression of IFN-γ, a crucial cytokine in Mtb resistance [51]. Furthermore, *B. fragilis* plays a role in the regulation of Th1 and Th2 balance, another major component of the immune response to Mtb infection [54]. In mice treated with orally administered *B. fragilis*, there was a decrease in tissue pathology and bacterial load in lungs, further highlighting the protective effects of increased lncRNA induced by *B. fragilis* [51]. *B. fragilis* can therefore positively influence host immune function to protect against Mtb.

Additionally, evidence suggests that *Heliobacter pylori* infection in humans and non-human primates is associated with immune protection against Mtb [55]. In an analysis conducted by Perry et al. of 339 human subjects, individuals with concurrent infection of latent TB and *H. pylori* had one and a half times higher production of IFN-γ than individuals with latent TB alone [55]. *H. pylori* may offer further protection by increasing immune Th1 response, which is recruited in response to Mtb infection [55,56]. Furthermore, Perry et al. evaluated cynomolgus macaques and found *H. pylori*-infected macaques showed a lower likelihood of progression to active TB than those not infected with *H. pylori* [55]. In humans, latent to active TB progression was less likely in *H. pylori*-infected individuals than in *non-H. pylori*-infected individuals within two years of exposure [55]. More research is therefore needed into how *H. pylori* attenuates TB progression and its potential role as a therapeutic remedy. The gut bacterial flora plays an important role in modulating immune function, and insight into the effects of dysbiosis on inflammatory processes can help to develop more effective Mtb treatments in the future.

## 5. Short-Chain Fatty Acids (SCFA)

SCFAs, particularly acetate, propionate, and butyrate, are major products of gut microbial fermentation [57]. SCFAs are transported into host cells and bind to G-protein coupled receptors (GPCR) on epithelial and immune cells and produce anti-inflammatory cytokines [31,37,58,59]. Butyrate, which can act in the lungs by increasing the phagocytic function of dendritic cells, has been associated with anti-inflammatory properties during Mtb infections by causing a decrease in the proinflammatory cytokines and inhibiting host response to the infection [60,61,62,63]. Butyrate may also be converted to Phenylbutyrate (PBA), which has a synergistic effect with vitamin D to inhibit Mtb growth [64]. This synergistic relationship upregulates LL-37, an antimicrobial peptide that increases the autophagy and intracellular killing of Mtb in host macrophages [62,65,66]. A study by Koh et al. hypothesizes the important regulatory role of SCFAs in T cell differentiation and function due to the high expression of SCFA receptors in immune cells; however, more research is required to determine the specific mechanism and role of SCFAs in TB therapy [59].

Interestingly, one study performed by Hu et al. analyzing stool samples of 46 TB patients and 31 healthy controls found that 9 out of 23 bacterial species enriched in healthy controls compared to TB patients were SCFA-producing bacteria such as *Roseburia inulinivorans*, *Bifidobacterium adolescentis*, *Ruminococcus obeum*, etc. [45,67]. Additionally, the butyrate-producing *Lachnospiraceae* and *Ruminococcaceae* families of the Firmicutes phylum were found to be decreased in individuals infected with Mtb [62]. Overall, TB patients saw a decrease in SCFA-producing bacteria in five pathways related to SFCA fermentation, thereby affecting inflammatory response and intestinal epithelial barrier strength [45,67]. Conversely, a study of fecal samples from TB patients by Maji et al. found SCFA-producers to increase in Mtb patients [68]. Given the anti-inflammatory roles of SCFAs and the potential for disease attenuation, additional research is needed to elucidate the impact of Mtb on SCFA-producers to better predict disease course and guide therapeutic regimens.

Furthermore, it is well established that DM is associated with an increased risk of Mtb infection [69]. High fat content diets and obesity, which can contribute to the progression of Type 2 DM, are associated with a decrease in Firmicutes and an increase in Bacteroidetes [61,70]. Many Firmicutes are involved in the production of SFCAs from the fermentation of insoluble fibers. In patients with Type 2 DM, SCFA-producing firmicutes such as *Faecalibacterium prausnitzii* are decreased [60]. The absence of these species was found to be associated with an increased incidence of low-grade inflammation in patients [60]. Additionally, the lack of *Lactobacillaceae* from high fat content diets is correlated with a strong inflammatory response [70]. The increased susceptibility to Mtb infection in patients with Type 2 DM may therefore be attributed to decreases in Firmicutes and the resultant decreases in SCFAs and increases in inflammation. Microbial alterations may therefore prove to be a reliable treatment option through enriching bacteria that amplify SCFA production and attenuate inflammatory processes to ease susceptibility in this patient population.

## 6. Effects of *Mycobacterium tuberculosis* on Bacterial Flora Composition

**Figure 3:**
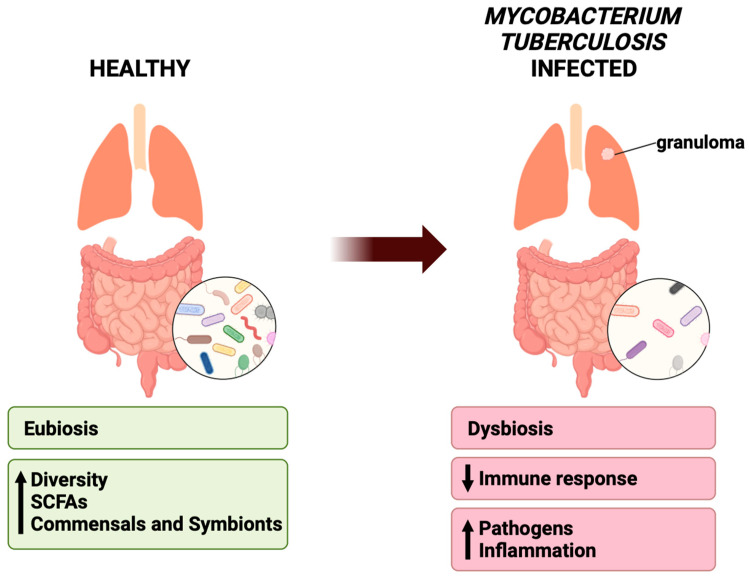
Healthy lungs can be characterized by eubiosis, with increased bacterial diversity, SCFA-producing bacteria, and commensals and symbionts. In *Mycobacterium tuberculosis* infected diseased lungs, there is a decreased immune response, increased pathogens and inflammation, and overall dysbiosis.

The microbiome can be altered by age, diet, antibiotics, disease, etc. [32,41,71,72,73,74]. Evidence suggests that upon infection by Mtb, the microbiomes in animal models and humans are susceptible to changes such as decreases in bacterial diversity as compared to healthy non-infected controls [33,41,44,45,54,67,71]. This change in bacterial composition could be attributed to physiological changes in the gut landscape such as inflammation and pH shifts, which may drive the out-competition of commensal strains by pathogenic strains [75,76]. This will subsequently alter metabolite production and immune function, which may further drive bacterial imbalance through enhancing inflammation and pathogenic growths (Figure 3) [77,78].

### 6.1. The Gut

In a study conducted by Luo et al., stool samples from 37 pulmonary TB patients and 20 healthy controls were obtained [41]. New TB patients were defined as subjects with newly developed pulmonary TB receiving less than or equal to one week of anti-TB treatment; recurrent TB patients were defined as subjects who had been previously treated and declared as clear prior to becoming bacteriologically positive again [41]. Individuals with a history of probiotic or antibiotic treatment for more than one week in the previous eight weeks were excluded [41]. Analysis of stool samples from all experimental groups demonstrated a decrease in the relative abundance of Bacteroidetes and to a lesser extent, Firmicutes, in TB patients as compared to healthy controls. TB patients also had an increase in Proteobacteria and Actinobacteria as compared to controls [41,42,43,44,45]. However, a greater increase in gut Proteobacteria was observed in new TB patients compared to recurrent TB patients [41]. This difference connects to studies from Sommer and Mori, who established that if the epithelial barrier is disturbed, the lipopolysaccharide component of the cell wall of the phylum Proteobacteria leads to activation of pro-inflammatory macrophages that trigger an inflammatory response both locally and at distant sites [71,79]. Additionally, evidence from animal models have shown that mice with gut colonization by *Helicobacter hepaticus* show difficulty controlling pulmonary mycobacterial growth when compared to non-infected mice [80].

In various studies, observed decreases were also seen in *Prevotella*, *Bacteriodes*, and the order of Clostridiales while significant increases were observed in *Escherichia* and *Streptococcus* in Mtb groups as compared to healthy controls [41,42,43,44,45,68]. Luo et al. additionally demonstrated that in individuals with recurrent Mtb infections, decreased *Prevotella* was linked to decreased CD4+ cells; in contrast, individuals with new Mtb infections had an increase in *Prevotella* that was positively associated with increased CD4+ cells [41].

Overall, Mtb induces a shift in the human bacterial profile, with notable decreases in Bacteroidetes and Firmicutes and increases in Proteobacteria. In order to develop microbiome-targeted therapies, more research is needed to elucidate additional relationships between Mtb and specific changes in bacterial composition as well as the roles of these changes in disease pathogenesis or attenuation.

### 6.2. The Lungs

Furthermore, recent evidence has shown that the lungs, once thought to be a sterile environment, is indeed an environment home to its own microbiome with differing microbial populations dependent upon location within the lungs and microbial immigration and elimination [74,81].

In healthy individuals, airways are predominantly populated by the phyla Firmicutes, Bacteroidetes, Actinobacteria, and Proteobacteria, with the common genera being *Prevotella, Streptococcus, Fusobacteria, Haemophilus,* and *Veillonella* [34,43,82,83,84,85,86]. In a study by Vázquez-Pérez et al. which utilized bronchoalveolar lavage (BAL) to compare the microbiota of six patients with active TB, six with pneumonia, and ten healthy controls, TB and pneumonia patients were found to have decreased diversity [85]. This study, in addition to others, found that in TB patients compared to healthy controls, bacterial taxa in the Actinobacteria phylum were found to be increased while taxa in the Firmicutes and Bacteroidetes phyla were found to be decreased [81,83,85,86]. Proteobacteria were also found to be increased in TB patients [85,87]; however, the opposite was observed as well [86]. In contrast, recent studies involving a meta-analysis and a multicenter analysis found no significant difference in diversity between pulmonary TB patients and healthy controls in the lower respiratory tract [81,83,88]. These discrepancies may be due to the lower biomass of the lung microbiome, which presents as a barrier to its accurate analysis [42,43], as well as limitations in data collection due to possible contamination with oropharyngeal flora [34,36,74,84,85,89,90,91,92,93]. More research is therefore needed to elucidate the changes Mtb imposes on microbial diversity.

Human studies of lung microbiomes using BAL have found that the dominant genus in TB patients was *Cupriavidus* compared to *Streptococcus* in healthy controls [85,89,94]. Studies using BAL fluid analysis show reduced microbial diversity and richness in TB patients compared to controls with unique genera found in each group [85,95]. A human study performed by Hu et al. analyzing BAL fluid found decreased alpha diversity in TB patients as well as significant differences in beta diversity between TB patients and healthy controls [96]. Mtb negative patients were observed to have enriched *Streptococcus*, *Prevotella*, *Nesseria, Bifidobacterium*, and *Selenomonas* compared to Mtb positive patients, which mirrors a healthy lung composition [96]. Lung microbiota containing oral commensals such as *Prevotella, Veillonella, and Streptococcus* lead to higher concentrations of metabolites such as arachidonic acid and pro-inflammatory phenotype characterized by the upregulation of IL-17 producing Th17 lymphocytes [97]. Thus, decreased microbial flora diversity in TB patients may be associated with altered and impaired immunity.

There is a growing body of evidence demonstrating the bidirectional interplay between the gut microbiome and lung microbiome, termed the gut-lung axis [32,34,43,98], and it is clear that Mtb infection has a dynamic impact on the microbiome. However, improvements in sampling technology and additional human studies must be performed to reliably elucidate the changes that occur within the human microbiome.

While the composition of the microbiome can contribute to disease alleviation, it can also potentially increase the risk of Mtb infection and hinder an individual’s ability to combat the disease effectively. It is widely acknowledged that the microbiota of TB patients differs from that of healthy individuals in terms of diversity, evenness, and abundance [99,100]. However, more research is necessary to comprehensively comprehend the modifications that can be made to the microbiome through various treatment regimens or dietary changes. These adjustments could potentially influence the growth of beneficial bacterial populations, reducing the risk of infection or aiding in the recovery from Mtb.

## 7. Antibiotic Treatments on Microbiome

Long-term use of antibiotics can cause dysbiosis of the gut microbiota through destroying both beneficial and pathogenic bacterial flora and by altering competition between species [48,101]. This microbial shift, which can last for up to three months or more after treatment course depending on medication type and duration, can confer susceptibility to Mtb and influence TB disease course by impairing host immune function [48].

In a study by Khan et al., mice were pre-treated with antibiotics and afterwards infected with Mtb while another group of mice was infected with Mtb and then post-treated with the same antibiotics; plus there was also a healthy control group and a group with Mtb-infection but no antibiotic treatment [47]. It was found that both groups of mice that received antibiotics experienced a higher Mtb burden in the lungs than the healthy and Mtb-without-antibiotics groups [47]. After five days, mice were more susceptible to Mtb infection after having antibiotics administered due to suppression of Th1 immunity [47]. Additionally, mice pre- and post- treated with antibiotics had larger and more numerous granulomas as compared to the Mtb-without-antibiotics mice group [47]. Before being sacrificed, some mice from both antibiotic treatment groups were given therapeutic fecal transplantation (FT) orally [47]. It was found that these mice with FT treatment had significantly less bacterial load in the lungs than their counterparts who did not receive FT [47]. Furthermore, according to a different study by Yang et al., the use of broad-spectrum antibiotic treatment increased susceptibility to Mtb and increased pulmonary inflammatory responses in Mtb-infected mice due to decreased *B. Fragilis* and lncRNA downregulation [49]. These studies suggest that microbial dysbiosis induced by antibiotics negatively impacts Mtb infection and TB disease course; however, partial microbial restoration with FT may present as a potential therapeutic option to aid in the fight against TB.

In a study conducted by Wipperman et al., individuals with Mtb treated for 6 months with isoniazid, rifampin, pyrazinamide, and ethambutol had significant decreases in the species *Ruminococcus*, *Eubacterium*, *Lactobacillus*, and *Bacteroides,* as well as an increase in *Erysipeloclostridium* and *Prevotella*. The *Ruminococcus* species plays a role in peripheral cytokine production, which includes IL-1 and IFN-γ. The *Bifidobacterium* species, a symbiotic bacteria found in the lamina propria of the small intestines, is found to increase Th17 response [48,102,103]. This suggests that the perturbations caused by Mtb treatment antibiotics have significant and long-lasting effects on immune responses [48].

Additionally, patients previously infected with Mtb are at an increased risk of re-infection than patients who have never been infected, as described in a study by Verver et al. [104]. In total, 612 patients who were reported TB positive between 1993–1998 were followed until 2021, and it was found that TB incidence in individuals previously successfully treated for TB was four times that of new TB cases. This could possibly be attributed to changes in gut bacterial composition and the resultant impact on host immune function. In a study by Luo et al., patients with recurring Mtb infections were found to have a significant difference in microbiota diversity than in patients with a new Mtb infection [41]. This alteration, potentially attributable to antibiotic treatment-induced bacterial shifts, could therefore impact and disturb peripheral immunity, increasing likelihood for reinfection [48]. Wu et al. conducted an analysis of sputum samples and throat swabs from 25 patients with new TB infections, including 20 who were cured after therapy, 30 recurrent TB patients who were declared cured prior to becoming bacteriologically positive again, 20 treatment failure patients who were smear positive after 5 months or more of treatment, and 20 healthy controls [105]. This study describes the abundance of the *Pseudomonas* genus in recurrent and treatment failure TB patients compared to new TB patients and healthy controls [105]. *Pseudomonas* is established to have a role in negatively contributing to disease processes in conditions such as cystic fibrosis and chronic obstructive pulmonary disease (COPD) [106,107]. The genera *Treponema* and *Atopobium* were also less abundant in recurrent TB patients than new TB patients and healthy controls [105]. Additionally, patients with recurrent TB infection had lower abundance of *Prevotella* compared to the other groups [41,105]. As described previously, *Prevotella* positively impacts metabolite concentration and immune function; therefore, its lesser abundance may contribute to compromised immunological function [41,97]. This implies that disruption in a balanced bacterial flora composition may pose as a risk factor for recurring TB infection.

There is sufficient evidence to suggest antibiotic treatments and previous Mtb infection lead to dysbiosis and susceptibility to Mtb, but further research into what particular changes in bacterial composition predisposes to Mtb reinfection is important for preventing and developing new treatment options for long-lasting protection against Mtb.

## 8. Future Therapies with *Mycobacterium tuberculosis*

Traditional medicine aims to alter a biochemical pathway involved in disease processes to restore normal function. Medical advances are opening the horizon for new and innovative treatment methods that might have been overlooked simply due to a lack of proper technology. Microbiome alteration and modification is one of many promising treatment options for chronic diseases, aging, neurodegenerative diseases, TB, and many more.

**Figure 4:**
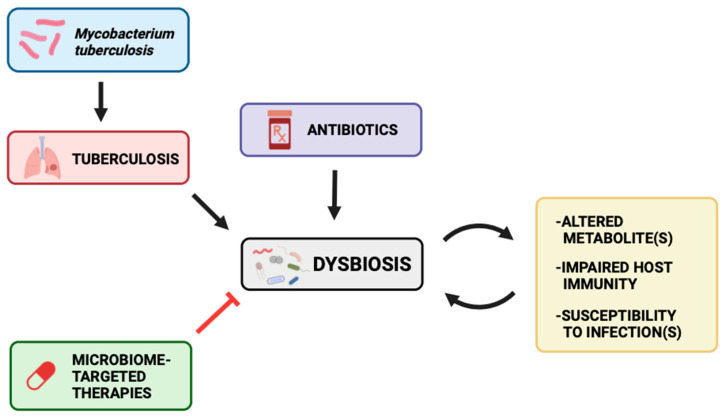
Mtb infection, consequent TB disease course, and antibiotics may alter the gut landscape and balance of commensals versus pathogens to give rise to dysbiosis. This may lead to altered metabolites, impaired host immunity, and susceptibility to infections. These may all further worsen the dysbiosis. Microbiome-targeted therapies, however, may be potential therapeutic options for TB disease course through attenuating the dysbiosis and its downstream effects.

The microbiome itself, through FT and diet alterations, can represent potential interventions to improve TB management and treatment. A longitudinal study performed by Wastyk et al. (2021) monitored microbiome diversity and immune status among healthy subjects who consumed either high-fiber or high-fermented foods for 10 weeks. Participants with the high-fermented food diet displayed increased species diversity and decreased inflammatory markers [37]. Additionally, Khan et al. notes how FT resulted in lower bacterial load in Mtb-infected mice that were treated with antibiotics compared to Mtb-infected mice that were not treated with antibiotics [47]. Recalling the gut-lung axis, high-fermented diets and FT offer potential avenues for TB therapy through increasing gut-protective bacteria abundance and anti-inflammatory mediators. This may therefore help restore the balance of commensal and pathogenic bacteria and positively influence metabolite production as well as host immune function (Figure 4). Furthermore, Yang et al. found that *B. fragilis* oral administration enhanced the expression of lncRNA-CGB which in turn promoted anti-TB immunity. This suggests that medically induced promotion of protective bacteria in the colon presents a promising venture for the development of Mtb-resistant hosts. The gut-lung axis has been implicated as a potential therapeutic target in disease courses; however, the underlying mechanism by which microbiota may impact TB outcomes is not clear [49]. More research is thus needed to elucidate the relationship between the microbiome and Mtb disease progression and immunity.

Targeting the microbiome may also provide relief and management for a variety of other diseases. Aging and neurodegenerative diseases are conditions that have potential to be addressed using microbiome-targeted therapies. Gut microbes may accelerate the development of neurodegenerative diseases by eliciting autoimmunity and producing metabolites; hence, gut microbes can be modulated to alleviate neurodegenerative diseases [108]. Based on available research, SCFAs may affect the brain through direct humoral effects, indirect hormonal effects, immune pathways, and neural pathways, and many psychological functions through interaction with G-protein coupled receptors or histone deacetylation [109]. According to Ho et al., in vitro selected SCFAs including butyric acid, valeric acid, and propionic acid inhibited amyloid beta aggregations, suggesting the potential use of SCFAs for treating Alzheimer’s Disease patients. Further studies need to be conducted to discern the effects of SCFAs on other neurodegenerative diseases including Parkinson’s and Huntington’s Disease [108]. Future studies need to focus on the utility of gut microbiome modification as a potential source of aging and neurodegenerative disease modification.

However, altering the gut microbiota as a therapeutic remedy is not without consequences. In a case study described by DeFilipp et al., the death of a patient through acquiring drug resistant *Escherichia coli* from FT therapy was described, and other complications such as ulcerative colitis flares and bacteremia have been reported [110,111,112]. Additionally, the use of probiotics and FT may result in abdominal bloating, abdominal distension, diarrhea, gas, and even brain fog [112,113]. Probiotics, in particular, are considered as foods or as dietary supplements rather than as drugs and are therefore subject to less stringent regulations. Lack of cohesiveness in safety, efficacy, and quality may pose as an additional hurdle to treatment success [114]. More consideration and caution should also be taken in certain populations prior to therapy initiation, notably in the immunocompromised and newborns because they may be at higher risk of developing adverse effects due to reduced abilities to clear microbiota [114]. Although this does not cover the full breadth of risks associated with microbe-altering treatments, the advantages and disadvantages must be further explored to minimize hazards and maximize safety while offering long-lasting protection against Mtb.

Considerable efforts are currently focused on understanding the nature of microbiome development and its effects on human health outcomes, most importantly the microbiome-molecular interactions and its complex mechanism of altering human pathophysiology. With technological advancements, additional calcification will be offered on the nature of human microbiome-pathophysiology interactions. Interventions focused on altering molecular-microbiome interactions will open new horizons and adventures in the field of medicine.

## Data Availability

Not applicable.
